# Genome Analysis of *Bifidobacterium Bifidum* E3, Structural Characteristics, and Antioxidant Properties of Exopolysaccharides

**DOI:** 10.3390/foods12162988

**Published:** 2023-08-08

**Authors:** Yingxue Yue, Yuqi Wang, Yu Han, Yifan Zhang, Ting Cao, Guicheng Huo, Bailiang Li

**Affiliations:** 1 Key Laboratory of Dairy Science, Ministry of Education, Northeast Agricultural University, Harbin 150030, Chinacao18088783239@163.com (T.C.);; 2 Food College, Northeast Agricultural University, Harbin 150030, China

**Keywords:** *Bifidobacterium bifidum*, genome, exopolysaccharide, antioxidant

## Abstract

In this study, the antioxidant properties of intact cells (IC), cell-free supernatant (CFS), and cell-free extracts (CFE) and whole genome sequencing of *Bifidobacterium bifidum* E3 (*B. bifidum* E3), as well as the structural characteristics and antioxidant properties of EPS-1, EPS-2, and EPS-3, were evaluated. The results revealed that intact cells (IC), cell-free supernatant (CFS), and cell-free extracts (CFE) had potent DPPH (1,1-Diphenyl-2-picrylhydrazyl radical), hydroxyl, and superoxide anion radical scavenging capacities, among which CFS was the best. At the genetic level, we identified a strong carbohydrate metabolism capacity, an EPS synthesis gene cluster, and five sugar nucleotides in *B. bifidum* E3. Therefore, we extracted cEPS from *B. bifidum* E3 and purified it to obtain EPS-1, EPS-2, and EPS-3. EPS-1, EPS-2, and EPS-3 were heteropolysaccharides with an average molecular weight of 4.15 × 10^4^ Da, 3.67 × 10^4^ Da, and 5.89 × 10^4^ Da, respectively. The EPS-1 and EPS-2 are mainly comprised of mannose and glucose, and the EPS-3 is mainly comprised of rhamnose, mannose, and glucose. The typical characteristic absorption peaks of polysaccharides were shown in Fourier transform infrared spectroscopy (FT-IR spectroscopy). The microstructural study showed a rough surface structure for EPS-1, EPS-2, and EPS-3. Furthermore, EPS-1, EPS-2, and EPS-3 exhibited potent DPPH, hydroxyl, and superoxide anion radical scavenging capacities. Correlation analysis identified that antioxidant capacities may be influenced by various factors, especially molecular weight, chemical compositions, and monosaccharide compositions. In summary, the EPS that was produced by *B. bifidum* E3 may provide insights into health-promoting benefits in humans.

## 1. Introduction

As Gram-positive bacteria, *Bifidobacterium* are an important member of the human gastrointestinal tract (GIT) microbiota and are part of the predominant gut microbiota of breastfed infants [1]. Some *Bifidobacterium* are safe, making their use as probiotics in pharmaceuticals and dairy products more common [2]. The purpose of studying the genomics of probiotics is to deeply understand the diversity and evolution of probiotics and try to uncover their molecular basis for health promotion [3]. *Bifidobacterium* are considered commensal microbes of the mammalian gut; many genes exist in their genomes, and these genes were predicted to encode enzymes [4]. In the perspective of genomics, genes involved in cEPS biosynthesis are usually clustered within specific genetic loci (commonly referred to as eps clusters) within members of the *Bifidobacterium* genus. Comparative genome analyses of different bifidobacterial species showed all analyzed taxa had at least one EPS locus, besides some *B. bifidum* strains [5].

Exopolysaccharides (EPS) are extracellular polymers that are commonly found in plants, animals, microorganisms, and other organisms. The EPS produced by lactic acid bacteria (LAB) has attracted widespread attention from people. As metabolites of probiotics, EPS can affect the flavor and texture of fermented products and the probiotic function [6]. It is reported that LAB-produced EPS have immune-enhancing, antioxidant, anti-cancer, anti-inflammatory, cholesterol-lowering, and antiviral effects [7]. The functional properties of polysaccharides (biological capacity and physicochemical properties) are closely linked with their structural characteristics (molecular weight (Mw), monosaccharide composition, glycosidic bonds, functional groups, and substituents, et al.) and complexity [8]. The relationship between EPS structure and its functional properties is currently not very clear. Therefore, it is crucial to characterize the chemical structures of LAB-EPS before exploring its functional properties.

Oxidative stress is caused by an imbalance between the production of oxidants and antioxidant defenses. Oxidative stress caused by excessive oxidation will accelerate aging, trigger inflammation, and reduce autoimmunity, thereby resulting in oxidative damage [9]. Exopolysaccharides that are isolated from lactic acid bacteria, such as *Lactiplantibacillus plantarum* (KX041), have antioxidant properties [10]. Inturri et al. [11] reported the chemical constituents of EPS produced by *Bifidobacterium longum* W11 and characterized its potential beneficial effects preliminarily, especially the antioxidant properties. Functional foods have attracted increasing attention, especially with the addition of probiotics to dairy products [12]. A study has shown that milk fermented with *Lactiplantibacillus plantarum* ATCC8014 has in vitro antioxidant properties with 14.7–48.9% DPPH capacity [13]. Therefore, it is very important to discover natural antioxidants that have no adverse impacts on human health.

The purpose of this study was to investigate the physical properties of EPS-1, EPS-2, and EPS-3 by ultraviolet-visible (UV-vis) spectrophotometry, FT-IR spectroscopy, congo red, and field emission scanning electron microscopy (FE-SEM). Meanwhile, the antioxidant capacities of IC, CFE, CFS, cEPS, EPS-1, EPS-2, and EPS-3 were also investigated. This study may help us further our understanding of the potential applications of functional foods and investigate the relationship between their structure and biological capacity.

## 2. Materials and Methods

### 2.1. Culture of Strain

*B. bifidum* E3 used in the current study was provided by Northeast Agricultural University (Harbin, China) and inoculated (3% *v*/*v*) into the MRS broth (Hopebio Company, Qingdao, China, HB0384-5) supplemented with 2% *v*/*v* L-cysteine hydrochloride (Biotopped Technology Co., Ltd., Beijing, China) at 37 °C for 24 h under the anaerobic condition. The strain was subcultured twice and then centrifuged (5000× *g*) for 10 min. The experimental supernatant was CFS. The precipitates from centrifugation were washed with PBS buffer three times, and *B. bifidum* E3 was resuspended with PBS (pH 7.4) buffer to achieve a final concentration of 1 × 10^9^ CFU/mL. The precipitates of the strains were the IC of the experiment. CFE was extracted by ultrasonic crushing of the IC. The treatment was performed under ultrasonic conditions at a power of 800 W for 3 s, stopped for 5 s, and continued for 15 min. All experimental samples were directly used for follow-up experiments.

### 2.2. Whole Genome Sequencing

The library was built from the DNA extracted from *B. bifidum* E3 and sequenced on the Illumina and PacBio RS II platforms. The Unicycler software was used to integrate and assemble data (https://github.com/rrwick/Unicycler (accessed on 20 October 2022)) and obtain the assembly results. The assembly results were aligned with known sequences in the NCBI to obtain the final assembled sequence. The genome circle map was drawn by CGView Server (http://cgview.ca/ (accessed on 22 October 2022)). KAAS (https://www.genome.jp/tools/kaas/ (accessed on 22 October 2022)) and WebMGA (http://weizhong-lab.ucsd.edu/webMGA/ (accessed on 22 October 2022)) websites were used to compare with the Kyoto Encyclopedia of Genes and Genomes (KEGG) and Cluster of Orthologous Groups (COG) of proteins to obtain the corresponding functional annotation information. The genome sequence was submitted to the National Center for Biotechnology Information (NCBI, Bethesda, MD, USA) database, and the NCBI prokaryotic genome annotation pipeline was used to annotate the whole genome (http://www.ncbi.nlm.nih.gov/books/NBK174280/ (accessed on 22 October 2022)).

### 2.3. Isolation and Purification of cEPS

The cold ethanol precipitation was used for EPS extraction [14]. Briefly, the CFS of *B. bifidum* E3 was inactivated in boiling water (100 °C) for 10 min and then cooled to room temperature. Proteins were precipitated by adding 80% (*w*/*v*) trichloroacetic acid (TCA) to the fermentation supernatant to achieve a final concentration of 4% (*w*/*v*). Furthermore, the above solution was incubated overnight at 4 °C and centrifuged at 5000× *g* for 20 min to obtain the supernatant. The supernatant was rotary evaporated and mixed with triple volumes of absolute ethanol to obtain the precipitate. Afterwards, the precipitate was dissolved in an appropriate amount of deionized water and dialyzed (8–14 kDa) at 4 °C for 48 h, followed by freeze-drying. Subsequently, the cEPS were purified by DEAE-Cellulose 52 eluting with deionized water and 0.1 and 0.3 mol/L NaCl at a flow rate of 1 mL/min, sequentially. Moreover, sephadex G-100 columns were used to elute NaCl (0.1 mol/L) at a flow rate of 0.5 mL/min to obtain EPS-1, EPS-2, and EPS-3.

### 2.4. Chemical Composition, Monosaccharide Composition, and Molecular Weight

The contents of neutral sugar, protein, uronic acid, and sulfate of EPS-1, EPS-2, and EPS-3 were determined according to the methods of phenol-sulfuric acid colorimetric [15], coomassie brilliant blue [16], sulfate-carbazole [17], and barium chloride-gelatin [18], with glucose, bovine serum albumin, galacturonic acid, and potassium sulfate as standards, respectively.

EPS-1, EPS-2, and EPS-3 were obtained according to Annadurai et al.’s method. [19]. The aqueous layer was filtered through 0.45-micrometer membranes. The quantitative analysis of monosaccharide composition was completed by the HPLC system (Agilent Technologies, Boeblingen, Germany). Acetonitrile and phosphate buffer (0.1 M, pH 6.7) were used as mobile phases (81:19) with a flow rate of 1.0 mL/min and an injection volume of 20 μL at a column temperature of 25 °C, and the detection wavelength was 245 nm. The monosaccharides of EPS-1, EPS-2, and EPS-3 were determined by measuring their retention time compared to standard sugars. Following that, the molar ratios of monosaccharide components were calculated.

Mw of EPS-1, EPS-2, and EPS-3 were determined by HPGPC according to the method of Mao et al. [20] with minor modifications. EPS-1, EPS-2, and EPS-3 (5 mg/mL) were centrifuged at 12,000 rpm for 10 min, and the supernatant was filtered through a 0.22 μm microporous membrane. The detection was performed with a RI-10A differential detector (Shimadzu, Kyoto, Japan), in which the column temperature was 40 °C, the injection volume was 20 μL, and the flow rate was 0.6 mL/min. Dextran MW standards from 1–670 kDa were used for the MW calibration.

### 2.5. Ultraviolet-Visible (UV-Vis) Spectrophotometry and Fourier-Transform Infrared (FT-IR) Spectroscopy

A UV-2600 spectrophotometer (Unico, Shanghai, China) was used to measure the absorption of EPS-1, EPS-2, and EPS-3 (1 mg/mL) from 190 to 500 nm. EPS-1, EPS-2, and EPS-3 (1 mg) were added with potassium bromide (KBr, 100 mg), then they were pressed into 1 millimeter-thick pellets for measurement. Moreover, an FT-IR spectrometer (Bruker Vetex70, Karlsruhe, Germany) was used to scan in the range of 4000–400 cm^−1^ [21].

### 2.6. Congo Red Test

The triple-helix structures of EPS-1, EPS-2, and EPS-3 were examined as follows: EPS-1, EPS-2, and EPS-3 (1 mg/mL) were dissolved in a NaOH solution (0.2 M) containing congo red (40 µM). To observe the changes in maximum absorption wavelength, we scanned the reaction solution in the spectral range of 190–800 nm [22].

### 2.7. Field Emission Scanning Electron Microscopes (FE-SEM)

A field emission scanning electron microscope (FE-SEM) (Hitachi S-4800, Hitachi, Tokyo, Japan) was used to analyze the apparent morphology of EPS-1, EPS-2, and EPS-3. In short, EPS-1, EPS-2, and EPS-3 were connected to an aluminum rod for gold sputtering and then observed at an accelerating voltage of 5 kV [23]. 

### 2.8. DPPH Radical Scavenging Assay

One milliliter of samples (IC, CFE, CFS, cEPS, EPS-1, EPS-2, and EPS-3) were added to 1 mL of DPPH (0.2 mmol/L), respectively. The mixture reacted in the dark at 37 °C for 30 min. After that, the supernatant was centrifuged at 5000× *g* for 10 min, and the absorbance was measured at 517 nm. The following is the calculation formula for radical scavenging [24]:

$$\mathrm{DPPH}\;\mathrm{radical}\;\mathrm{scavenging}\;(\%)=(1\;-\;\frac{Sample\;OD517-Blank\;OD517}{Control\;OD517})\;\times \;100$$

In the formula, DPPH solution dissolved in anhydrous ethanol was added to the blank group, while PBS was used to replace the sample in the control group, and other reaction conditions were the same.

### 2.9. Hydroxyl Radical Scavenging Assay

The hydroxyl radical scavenging test kit (No. A018-1-1; Nanjing Jiancheng Bioengineering Institute, Nanjing, China) was used to determine the hydroxyl radical scavenging capacity of samples (IC, CFE, CFS, cEPS, EPS-1, EPS-2, and EPS-3). The following is the calculation formula for radical scavenging:

$$\mathrm{Hydroxyl}\;\mathrm{radical}\;\mathrm{scavenging}\;\mathrm{capacity}\;(\%)=\frac{Control\;OD550-Sample\;OD550}{Control\;OD550-Blank\;OD550}\;\times \;100$$

### 2.10. Superoxide Anion Radical Scavenging Assay

The reaction solution contained 2.8 mL of Tris-HCl (0.05 mol/L, pH 8.2), 0.1 mL of pyrogallol (0.05 mol/L), and 0.1 mL of IC, CFE, CFS, cEPS, EPS-1, EPS-2, and EPS-3 at room temperature to avoid light reactions for 4 min. Furthermore, 1 mL of hydrochloric acid (8 mol/L) terminated the reaction, and the absorbance of the final solution was measured at 320 nm [10]. The following is the calculation formula for radical scavenging:

$$\mathrm{Superoxide}\;\mathrm{anion}\;\mathrm{radical}\;\mathrm{scavenging}\;\mathrm{capacity}\;(\%)=(1\;-\;\frac{Sample\;OD320}{Blank\;OD320})\;\times \;100$$

Deionized water was used instead of samples in the blank group.

### 2.11. Statistical Analysis

All data were exhibited as mean ± standard deviation (*n* = 3) and analyzed by one-way analysis of variance (ANOVA) using SPSS 18.0 software (SPSS Inc., Chicago, IL, USA). Graphs were drawn with GraphPad Prism 8.0.2 (GraphPad Software, La Jolla, CA, USA). In all results, *p* < 0.05 was considered to be a significant difference, and *p* > 0.05 was considered not to be a significant difference.

## 3. Results and Discussion

### 3.1. Antioxidant Capacity of IC, CFE, and CFS

**Figure 1:**
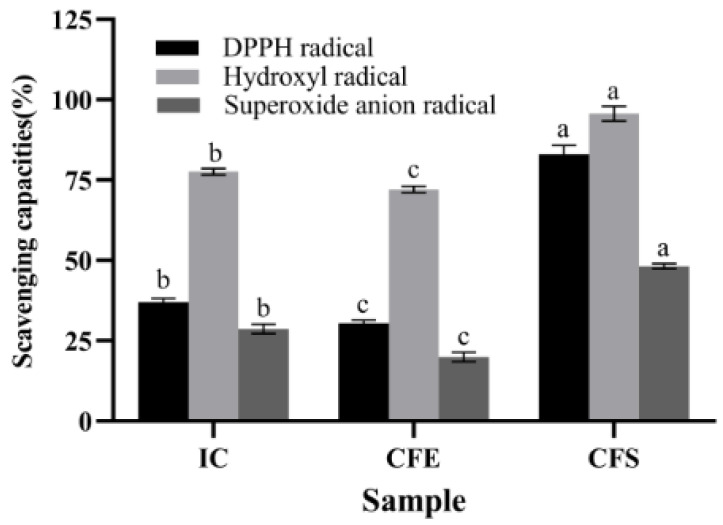
Antioxidant capacities of IC, CFE, and CFS. Values with different superscript letters (a, b, and c) significantly differed at *p* < 0.05 in the same group.

The antioxidant capacities of IC, CFE, and CFS were demonstrated in Figure 1. The scavenging capacities of IC, CFE, and CFS for DPPH radical, hydroxyl radical, and superoxide anion radical ranged from 18.33% to 98.07%. CFS had the highest scavenging capacities with 83.08% ± 2.02%, 95.68% ± 1.60%, and 48.20% ± 0.55% for DPPH radical, hydroxyl radical, and superoxide anion radical, which were significantly higher than IC and CFE (*p* < 0.05). The scavenging capacities of DPPH radical, hydroxyl radical, and superoxide anion radical of IC were 37.07% ± 0.78%, 77.63% ± 0.74%, and 28.72% ± 1.11%. Meanwhile, the three scavenging capacities of IC were significantly higher than those of CFE (*p* < 0.05). Ma et al. [25] found that CFS of *Bifidobacterium infantis* YLGB-1496 scavenged 41.18%, 102.38%, and 24.88% of DPPH radical, hydroxyl radical, and superoxide anion radical, respectively. The scavenging capacity of *B. bifidum* E3 was higher than that of *Bifidobacterium infantis* YLGB-1496 for DPPH radical and superoxide anion radical. Zhao et al. [26] found that *Bifidobacterium longum subspecies* exhibited free radical scavenging ability, especially CFS, which showed higher antioxidant capacity than IC and CFE, which was consistent with our study. The capacity of CFS to scavenge radicals may be related to the metabolites. Exopolysaccharide is one of the metabolites in CFS. Therefore, we focused on analyzing the basic genomic information of *B. bifidum* E3, especially in the genes of its sugar transport system and the cluster of exopolysaccharides.

### 3.2. Analysis of the Genome 

#### 3.2.1. Genome Composition

**Figure 2:**
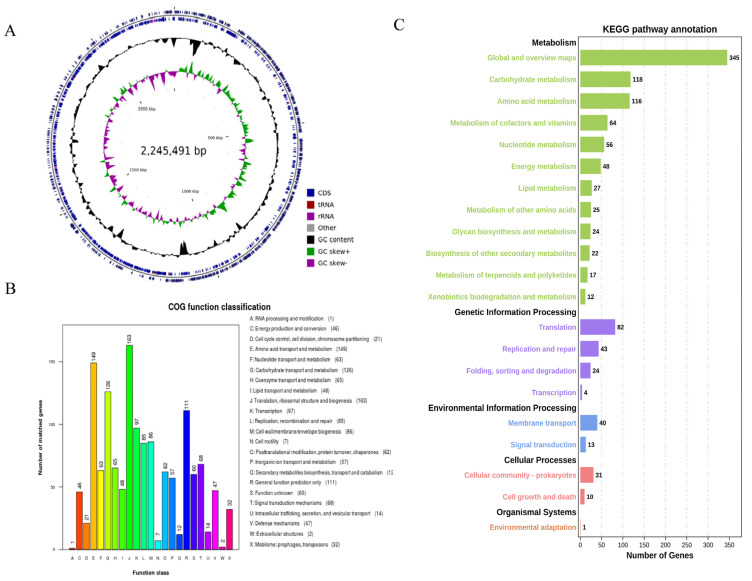
Circular genome map of *B. bifidum* E3 (**A**) COG functional classification of the *B. bifidum* E3 genome (**B**) KEGG pathway classification of the E3 genome (**C**) From the outer circle to the inner circle, the first circle and the second circle represent genes on the positive and negative strands, including CDS, tRNA, rRNA, and other genes; the third circle represents GC content; the fourth circle represents GC skew; where green means GC > 0, and purple means GC < 0, the junction of green and purple is the start point and end point of replication, respectively.

The genome of *B. bifidum* E3 had no plasmids and only consisted of a circular chromosome (2,245,491 bp) with a G + C content of 62.75%. The genome predicted 1878 genes with a total length of 1,945,738 bp, and the total length of the coding regions was 86.65% of the whole genome. Among them, the average length of coding genes was 1036 bp. In addition, 1818 CDSs were predicted in the genome. There were 53 tRNAs, 1 tmRNA, and 6 rRNA (16 s and 23 s) gene operons without sRNA (Figure 2A).

#### 3.2.2. Analysis of COG and KEGG

The result of COG annotation is shown in Figure 2B. There were 1422 genes that could be assigned to COG families, comprising 23 functional categories. There were 1, 46, 21, 149, 63, 126, 65, 48, 163, 97, 85, 86, 7, 62, 57, 12, 111, 60, 68, 14, 47, 2, and 32 genes annotated to categories A to X, respectively. Among them, a large number of genes were annotated in four categories: translation, ribosomal structure, and biogenesis (11.46%), amino acid transport and metabolism (10.48%), carbohydrate transport and metabolism (8.86%), and general function prediction only (7.81%).

The classification of genes that code for proteins in the KEGG pathway in the genome of *B. bifidum* E3 was obtained through KAAS annotation (Figure 2C). A total of 1122 genes were identified, assigned to 116 KEGG pathways, and divided into five categories. The pathways with the most annotated genes were global and overview maps (30.75%), carbohydrate metabolism (10.52%), and amino acid metabolism (10.34%). Secondly, more genes were involved in transcription (7.31%) and the metabolism of cofactors and vitamins (5.70%). Therefore, it was important to understand the relevance of carbohydrate metabolism to function. Next, we investigated the synthesis of exopolysaccharides from a genomic perspective as well as their relationship with in vitro antioxidant capacities.

### 3.3. Sugar Transport System 

In general, carbohydrates are transported in three ways: phosphotransferase systems (PTS), sugar penetration, and ABC-type sugar transfer systems. The PTS system is the main transport method and consists of histidine phosphate carrier protein (HPr), enzyme I (EI), and sugar-specific enzyme II (EII). The analysis of the sugar transport system of *B. bifidum* E3 is shown in Table 1. In the genome, genes E3_01768 and E3_01767 encode HPr and EI. *B. bifidum* E3 had 4 EIIs related to the PTS system. They were EIIB of transport sugar (E3_01751), EIIA of glucose (E3_00280), EIIABC of N-acetylglucosamine (E3_00281), and EIIABC of fructose (E3_01538). One gene (E3_01046) encoded the permease of the ABC-type sugar transport system, and three genes (E3_01045, E3_01047, and E3_01393) encoded multiple sugar transport system permease proteins in the genome of *B. bifidum* E3. In addition, the galactose permease encoded by gene E3_01493 could transport lactose, the fructose permease encoded by gene E3_01538 could transport fructose, and the lactose permease encoded by gene E3_01046 could transport lactose. The above analysis showed that *B. bifidum* E3 could transport lactose, fructose, glucose, N-acetylglucosamine, and galactose, which formed the substrate for the synthesis of sugar nucleotides and activated precursors for EPS synthesis.

### 3.4. Synthesis of Sugar Nucleotides and Gene Cluster

**Figure 3:**
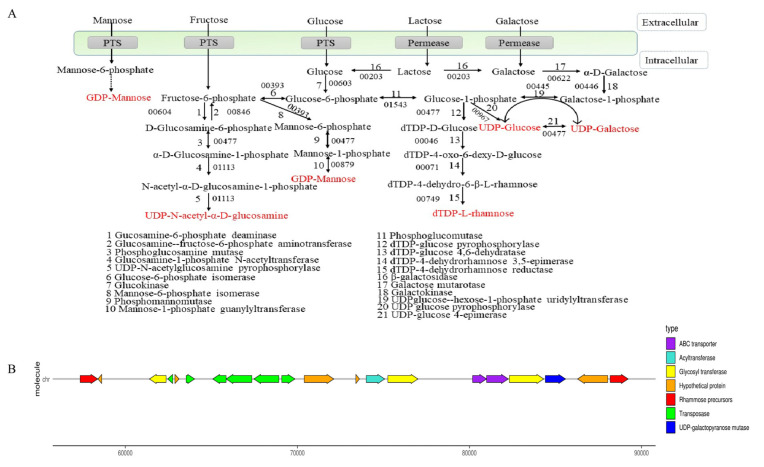
Synthesis of sugar nucleotides (**A**) and gene clusters (**B**) of *B. bifidum* E3.

The synthesis process of EPS is divided into two stages: the synthesis of precursor sugar nucleotides and EPS gene clusters. As shown in Figure 3A, lactose could be hydrolyzed into glucose and galactose by beta-galactosidase (E3_00203). Galactose was transformed into glucose-1-phosphate through galactokinase (E3_00446) and UDP-glucose-hexose-1-phosphate uridylyltransferase (E3_00445). Glucose was transported into the cell by the transport system, and glucose-6-phosphate was formed by the action of glucokinase (E3_00603). Partial glucose 6-phosphate was transformed into glucose 1-phosphate by phosphoglucomutase (E3_01543) and formed UDP-glucose through UDP glucose pyrophosphorylase (E3_00967). UDP-galactose and UDP-glucose could be converted into each other in the presence of UDP-galactose-4-epimerase (E3_0047). Glucose-1-phosphate could also be catalyzed by dTDP-glucose 4,6-dehydratase (E3_00046), dTDP-4-dehydrorhamnose 3,5-epimerase (E3_00071), and dTDP-4-dehydrorhamnose reductase (E3_00749) to synthesize dTDP-L-rhamnose. The PTS system transported fructose into the cytoplasm, and then fructose-1-phosphate was converted to fructose-6-phosphate by glucosamine-6-phosphate deaminase (E3_00604). Fructose-6-phosphate was transformed into UDP-N-acetyl-α-D-glucosamine through phosphoglucosamine mutase (E3_00477), glucosamine-1-phosphate N-acetyltransferase (E3_01113), and UDP-N-acetylglucosamine pyrophosphorylase (E3_01113). Meanwhile, fructose-6-phosphate was converted to GDP-mannose under mannose-6-phosphate isomerase (E3_00393), phosphomannomutase (E3_00477), and mannose-1-phosphate guanylyltransferase (E3_00879). These nucleotide sugars might be assembled into the repeating units of EPS.

Most EPS-producing microorganisms have genes encoding for the synthesis of EPS clustered in their genomes or large plasmids. As shown in Figure 3B, *B. bifidum* E3 had one EPS synthesis gene cluster consisting of 20 genes. The cluster of EPS genes was related to the encoding of transposase (E3_00050, E3_00052, and E3_00054-57), rhamnose precursors (E3_00046 and E3_00071), ABC transporters (E3_00065 and E3_00066), acyltransferase (E3_00060), UDP-galactopyranose mutase (E3_00068), and glycosyl transferase (E3_00049, E3_00061, and E3_00067). Glycosyl transferase plays a crucial role in determining the monosaccharide composition of EPS [27]. Transposase facilitates the stabilization of exopolysaccharide gene clusters, which indicates the possibility of horizontal gene transfer between *B. bifidum* E3 and other strains. The effect of these genes on the chemical structures and functions of *B. bifidum* E3 EPS remains to be determined. Meanwhile, we also found a large number of hypothetical proteins in the gene cluster. Furthermore, we investigated the physical properties and antioxidant capacities of the exopolysaccharide of *B. bifidum* E3.

### 3.5. Antioxidant Capacity of cEPS

**Figure 4:**
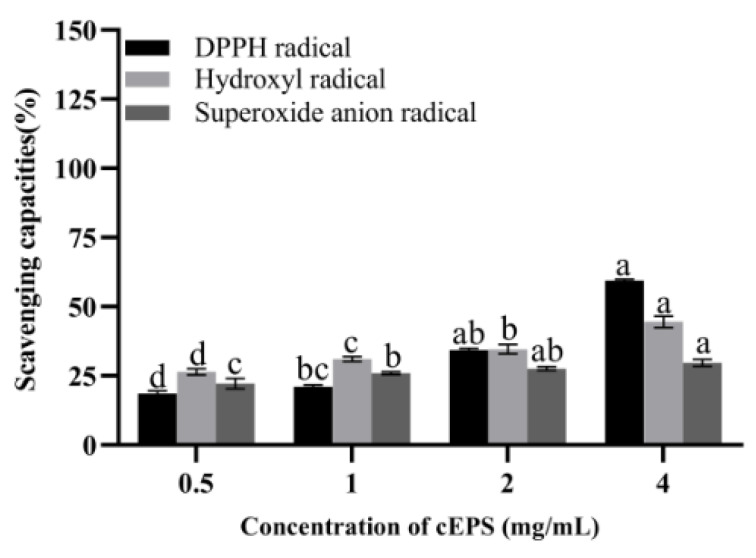
Antioxidant capacities of cEPS. Values with different superscript letters (a, b, c and d) significantly differed at *p* < 0.05 in the same group.

The antioxidant properties of cEPS from the *B. bifidum* E3 metabolite are shown in Figure 4. It was found that the scavenging capacities of DPPH radical, hydroxyl radical, and superoxide anions of cEPS significantly increased with increasing cEPS concentration, respectively. The antioxidant property of cEPS (4 mg/mL) was the highest compared to the concentration of 0.5–2 mg/mL. The scavenging abilities of DPPH and superoxide anions radicals of cEPS (4 mg/mL) were significantly different at concentrations of 0.5 and 1 mg/mL (*p* < 0.05). Similarly, the scavenging ability of hydroxyl radicals at each cEPS concentration was significantly different (*p* < 0.05). cEPS may have strong radical scavenging capacities due to its antioxidant components, such as protein and trace elements. Li et al. [28] found the antioxidant properties of cEPS from *Lactococcus lactis* subsp. *lactis* IMAU11823 were higher than their purified fractions, and cEPS had excellent antioxidant properties, which was consistent with our study. As a result, cEPS might be beneficial to the reduction of oxidative stress caused by substances, such as radicals and reactive oxygen species, with the potential to be a natural antioxidant.

### 3.6. Yield, Chemical Compositions, Monosaccharide Compositions, and Molecular Weight

The basic components of EPS-1, EPS-2, and EPS-3 are presented in Table 2. The cEPS yield of *B. bifidum* E3 was 429.78 mg/L. A study indicated that the greatest EPS yield of *Bifidobacterium bifidum* WBIN03 was 241.20 mg/L in MRSc medium [29]. Therefore, *B. bifidum* E3 produced a much higher level of cEPS compared with *Bifidobacterium bifidum* WBIN03. Three components were obtained by purifying cEPS, and the purification rates of EPS-1, EPS-2, and EPS-3 were 29.33%, 23.00%, and 10.67%, respectively. The final yields of *Lactobacillus rhamnosus* ZFM231 were 18.65% (EPS-1), 22.52.0% (EPS-2), 16.75% (EPS-3), and 17.58% (EPS-4), respectively [30]. The EPS-1 and EPS-2 yields of *B. bifidum* E3 were higher than those of *Lactobacillus rhamnosus* ZFM231, while the yield of EPS-3 was lower than that of *Lactobacillus rhamnosus* ZFM231. The neutral sugar constituted a major proportion in EPS-1, EPS-2, and EPS-3: 90.53 ± 0.52%, 89.16 ± 1.34%, and 72.47 ± 2.81%, respectively. In contrast, the protein content of EPS-1, EPS-2, and EPS-3 was low. The uronic acid contents of EPS-1, EPS-2, and EPS-3 were significantly different from each other (*p* < 0.05), and EPS-3 had the highest content of 0.94 ± 0.07. The sulfate contents of EPS-2 and EPS-3 were not significantly different (*p* > 0.05), but they were significantly different (*p* < 0.05) when compared with EPS-1. The EPS produced by *L. helveticus* MB2-1 contained 5.24% and 0.47% of uronic acid and sulfate, respectively [31]. The uronic acid contents of EPS-1, EPS-2, and EPS-3 obtained in our experiment were lower than in situ EPS produced by *L. helveticus* MB2-1, but the sulfate contents of them were higher than *L. helveticus* MB2-1.

The monosaccharide composition of EPS-1, EPS-2, and EPS-3 produced by *B. bifidum* E3 was investigated by HPLC. The analysis results for the monosaccharide composition are shown in Table 2. EPS-1 was mainly comprised of mannose and glucose, and its molar ratio was approximately 1:5.04. EPS-2 was mainly comprised of rhamnose, mannose, and glucose; its molar ratio was approximately 0.32:1:1.56. EPS-1 produced by *Lactococcus lactis* subsp. *lactis* IMAU11823 was composed of glucose and mannose with a molar ratio of 7.01:1.00, whereas EPS-2 was composed of mannose, glucose, and rhamnose with a molar ratio of 7.45:1.00:2.34 [28]. The monosaccharide composition was consistent with our study. ESP-3 was mainly comprised of rhamnose, mannose, glucosamine, galactose, glucose, and glucuronic acid; its molar ratio was approximately 1.10:1:0.82:1.05:1.39 and 0.68, suggesting that it could be an acidic polysaccharide. There are few reports on the monosaccharide composition of bacterial polysaccharides. However, it was found in a study that EPS364 (charged exopolysaccharide of novel deep-sea bacteria) was composed of mannose, glucosamine, glucose, galactosamine, and arabinose in a molar ratio of 5:9:3.4:0.5:0 [32]. Studies indicated that polysaccharides containing mannose in the composition of monosaccharides had the potential function of lowering blood sugar and inhibiting the growth of tumors [33]. The above results showed that the species of strains might affect the monosaccharide composition and proportion of EPS. 

It has been reported that the molecular weight range of EPS in lactic acid bacteria is 10^4^–10^6^ Da [34]. The molecular weights of EPS-1, EPS-2, and EPS-3 in this experiment were 4.15 × 10^4^ Da, 3.6 × 10^4^ Da, and 5.89 × 10^4^ Da, respectively. Molecular weight is an essential property of EPS that may affect probiotics. High-molecular-weight EPS is stronger than low-molecular-weight EPS in improving the texture of fermented milks and their anti-tumor activity [35]. Furthermore, the differences between Mws of different EPSs may be related to their specific functions.

### 3.7. UV-Vis Spectrophotometry and FT-IR Spectroscopy

**Figure 5:**
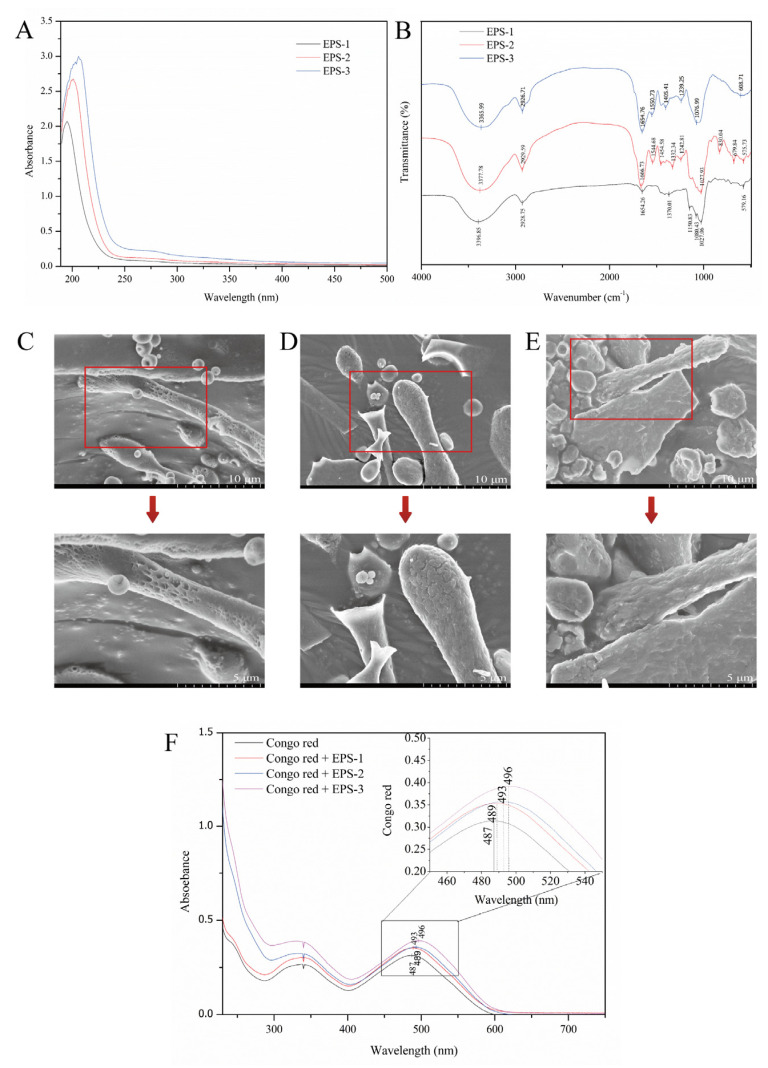
Ultraviolet spectroscopy (**A**) and FT-IR spectroscopy (**B**) of EPS-1, EPS-2, and EPS-3; Field emission scanning electron microscopes of EPS-1 (**C**), EPS-2 (**D**), and EPS-3 (**E**) at magnifications of 5000× and 10,000×; Congo red determination of EPS-1, EPS-2, and EPS-3 (**F**).

To verify whether EPS-1, EPS-2, and EPS-3 contain nucleic acids and proteins, the three components were scanned by the UV-vis spectrum in the wavelength range of 190–500 nm. The results showed that there was only one absorption peak around 200 nm. The three components had no absorption peaks at 260 nm and 280 nm wavelengths (Figure 5A). Therefore, there were no nucleic acids and very little protein in the three components.

FT-IR spectroscopy is a useful analytical method for studying biopolymers’ structures and functional groups. The FTIR spectra of EPS-1, EPS-2, and EPS-3 are shown in Figure 5B. EPS-1, EPS-2, and EPS-3 had characteristic absorption peaks of typical polysaccharides, such as 3396.85 cm^−1^, 3377.78 cm^−1^, and 3365.99 cm^−1^, which were associated with the carbohydrate hydroxyl groups. Many hydroxyl groups are shown at peaks of 3200–3600 cm^−1^. The peaks at 2928.75 cm^−1^, 2929.59 cm^−1^, 2926.71 cm^−1^, and 1654.26 cm^−1^, 1666.73 cm^−1^, and 1654.76 cm^−1^ in the three components were due to the stretching vibration of C-H and C = O groups in the sugar ring [36]. The absorbance in the 1700-1550 cm^−1^ region may be due to the presence of uronic acid. The peak at 1370.01 cm^−1^ and 1332.34 cm^−1^ in EPS-1 and EPS-2 might be attributable to the stretching vibration of -CH3 [37]. Some weak stretching peaks at 1242.81 cm^−1^, 1239.25 cm^−1^ in EPS-2 and EPS-3, and the sharp peaks at 1027.06 cm^−1^, 1027.93 cm^−1^, and 1076.99 cm^−1^ in EPS-1, EPS-2, and EPS-3 suggested the existence of carboxylic acids and C-O stretching vibrations of ester groups [38]. Ayyash et al. indicated that the wave number region 1250-1000 cm^−1^ is attributed to the existence of carbohydrates, and peaks in this region belong to C-O-C and C-O-H glycosidic bands [39]. Additionally, the peak at 830.04 cm^−1^ in EPS-2 might indicate the presence of a mannose ring, which is consistent with monosaccharide composition. Weak peaks observed at 679.84 cm^−1^ and 608.70 cm^−1^ in EPS-2 and EPS-3 demonstrated the variation of the C-H bend group at 700–600 cm^−1^. The weak peaks at 579.16 cm^−1^ and 575.73 cm^−1^ might result from sulfate groups, which were in accordance with the result of Di et al. [40] These results indicated that the FT-IR spectroscopy of EPS-1, EPS-2, and EPS-3 had the main characteristics of polysaccharides.

### 3.8. Field Emission Scanning Electron Microscope

FE-SEM is a helpful tool for studying the surface and three-dimensional morphologies of biological macromolecules and helps to understand the physical properties of polysaccharides. The apparent morphology and physicochemical properties of polysaccharides were observed by FE-SEM and shown in Figure 5C-E. The additional details of the EPS microstructure can be seen after the microscopic images at 10,000× magnification, showing a rough and lumpy surface. A dense and porous sponge structure composed of irregularly shaped particles was observed in EPS-1, giving the polysaccharide water retention capacity. The MSR101 strain had a similar structure to that of EPS-1 [41]. EPS-2 had many uniform flake and rod-shaped structures, revealing a compact structure with a rough surface. The microscopic morphology of EPS-3 showed a block-like structure with an uneven surface. It was similar to the appearance of the exopolysaccharide produced by Tibetan kefir grains during the fermentation of milk [42]. The differences in morphology between EPS-1, EPS-2, and EPS-3 might be related to the composition and structure of monosaccharides. 

### 3.9. Colorimetric Determination of Triple Helix Structures

In the weak alkaline solution, polysaccharides with tripartite helix structures will form complexes with congo red. The complexation is related to a bathochromic shift in the maximum absorption wavelength of the congo red spectrum. The results showed that EPS-1 and EPS-2 have no notable bathochromic shift, indicating the absence of a triple helix arrangement (Figure 5F). Polysaccharides extracted from *L. mesenteroides* DRP105 showed the same conformation [43]. However, the maximum absorption wavelength difference between EPS-3 + congo red and congo red was 9 nm. It indicated that EPS-3 might have a triple helix structure. It was consistent with our study that exopolysaccharide (cEPS) produced by *Lactobacillus helveticus* MB2-1 exhibited a significant bathochromic shift of approximately 20 nm in the λmax [22]. Triple helices in polysaccharides might be associated with biological capacity.

### 3.10. Antioxidant Capacity of EPS-1, EPS-2, and EPS-3 

**Figure 6:**
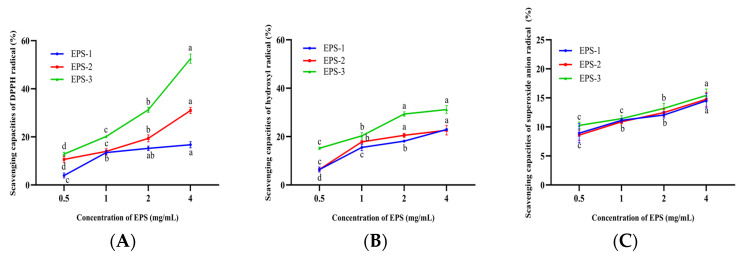
Scavenging capacities of EPS-1, EPS-2, and EPS-3 on DPPH radicals (**A**), hydroxyl radicals (**B**), and superoxide anion radicals (**C**). Values with different superscript letters (a, b, c and d) significantly differed at *p* < 0.05 in the same group.

The antioxidant capacities of EPS-1, EPS-2, and EPS-3 showed dose-dependent responses with increasing concentration, and EPS-3 exhibited the greatest capabilities in scavenging radicals (Figure 6A–C). The scavenging capacities of DPPH radicals in EPS-2 and EPS-3 were significantly increased from 2.8% to 53.64% at a concentration of 0.5–4 mg/mL (*p* < 0.05). However, there were no significant differences at concentrations of 1–4 mg/mL of EPS-1 (*p* > 0.05). The scavenging DPPH radical abilities of ST-EPS1, ST-EPS2, and ST-EPS3, which are produced by *Streptococcus thermophilus* CGMCC 7.179, were 47.11%, 70.18%, and 41.79% at 10 mg/mL, respectively [44]. EPS-3 (4 mg/mL) of *B. bifidum* E3 was higher than ST-EPS1 and ST-EPS3 on the scavenging capacity of DPPH radicals. DPPH radicals are considered stable radicals that maintain their stability by accepting electrons or hydrogen groups, which means that EPS-1, EPS-2, and EPS-3 might be good electron or hydrogen acceptors.

The scavenging capacities of hydroxyl radicals in EPS-1, EPS-2, and EPS-3 were significantly increased at concentrations of 0.5–2 mg/mL (*p* < 0.05). The hydroxyl radical scavenging capacities were 22.95 ± 0.26%, 22.62 ± 1.93%, and 31.15 ± 1.60% for EPS-1, EPS-2, and EPS-3 at 4.0 mg/mL. Xu et al. found the acidic polysaccharide of *Bifidobacterium animals* RH had better hydroxyl radical scavenging ability than neutral polysaccharide [45]. In this experiment, the hydroxyl radical scavenging capacities of neutral polysaccharides (EPS-1 and EPS-2) were lower than those of acidic polysaccharides (EPS-3), which was consistent with the previous report. The above studies could suggest that EPS-1, EPS-2, and EPS-3 might help to mitigate cellular oxidative damage caused by hydroxyl radicals and alleviate the degree of chronic disease in humans.

EPS-1, EPS-2, and EPS-3 showed significant differences between the concentrations of 0.5 and 4 mg/mL and achieved the maximum superoxide anions radical scavenging capacities (14.50 ± 1.24%, 14.71 ± 1.7%, and 14.89 ± 0.88%) when the concentration was 4 mg/mL, respectively. The scavenging of superoxide anion radicals by EPS was directly correlated with the concentration. However, three components showed little difference in scavenging at the same concentrations. Liu et al. found that the acidic polysaccharide of the purified fraction of *Saccharomyces cerevisiae* Y3 had significant radical scavenging abilities, with a scavenging rate of 18.80% [46], which was similar to the superoxide anion scavenging rate of EPS-3 (15.42 ± 0.77%). The above research showed that EPS had a superior capacity to scavenge superoxide anion radicals. Therefore, EPS is important for mitigating oxidative stress and oxidative damage.

### 3.11. Correlation Analysis 

**Figure 7:**
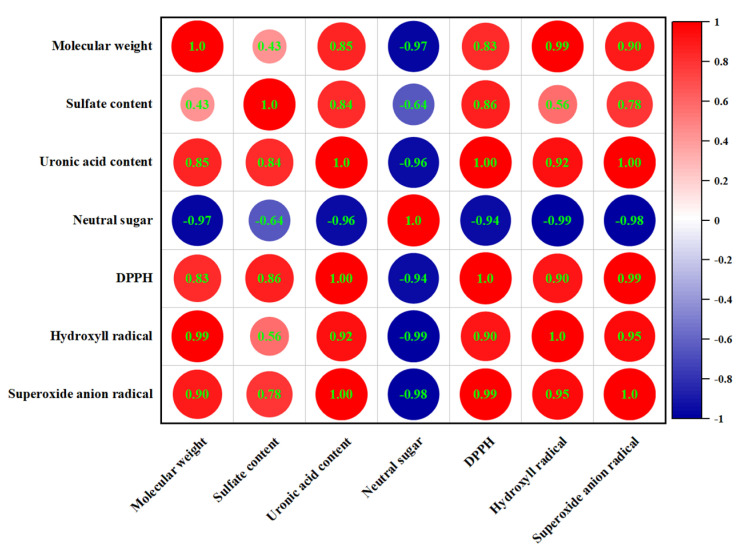
Correlation between chemical composition and antioxidant capacities.

The Spearman correlation analysis was used to demonstrate the correlations between the chemical composition, molecular weight, and antioxidant capacities of EPSs (Figure 7). There was a negative correlation between the neutral sugar content and antioxidant capacities. In contrast, uronic acid and sulfate contents showed a positive correlation with DPPH, hydroxyl, and superoxide anion radical scavenging capacities; the correlation coefficients were 1.00, 0.92, 1.00, and 0.86, 0.56, and 0.78, respectively. Meanwhile, we found a similar positive relationship between molecular weight and antioxidant capacity. It has been suggested that the strong antioxidant capacities of polysaccharides from cyclina sinensis (CSPS) may be due to their high content of uronic acid, protein, and sulfate [47]. Wang et al. showed that exopolysaccharides with higher sulfate content have higher antioxidant activity [48]. These results were the same as the results of this experiment. In addition, the antioxidant capacities are also closely related to the molecular weight. Exopolysaccharides with a higher molecular weight have larger organic groups than polysaccharides with a lower molecular weight, which contribute to increased antioxidant activity [49]. However, other studies have found that low-molecular-weight EPS can also have high antioxidant capacity [50]. Therefore, the effect of molecular weight on antioxidant properties was complex. The monosaccharide composition of EPS also affects its antioxidant capacity. We explored the correlation between them using multiple linear regression analysis and showed it in the following formula:

DPPH radical scavenging capacity (%) = 3.696 + 0.786 Xmannose + 3.182 Xglucuronic acid

Hydroxyl radical scavenging capacity (%) = 22.319 + 0.018 Xmannose + 0.758 Xglucuronic acid

Superoxide anion radical scavenging capacity (%) = 14.279 + 0.014 Xmannose + 0.081 Xglucuronic acid

The results showed that the increase in DPPH, hydroxyl, and superoxide anion raical scavenging capacities was related to the increase in mannose and glucuronic acid. The positive coefficients in front of Xglucuronic acid were higher than Xmannose, indicating that the presence of glucuronic acid has stronger radical scavenging capacities than mannose. A high molar ratio of mannose in exopolysaccharides extracted from inonotus obliquus enhanced antioxidant capacities [51]. The presence of uronic acid groups could increase the negative charge of polysaccharides on the one hand and effectively scavenge free radicals by activating hydrogen atoms on the other [52]. Thus, it appeared that the antioxidant capacities were attributed to several factors.

## 4. Conclusions

In the present study, it was found that IC, CFE, and CFS of *B. bifidum* E3 had potent antioxidant capacities, among which CFS had the highest antioxidant capacity. Meanwhile, *B. bifidum* E3 showed superior performance in carbohydrate metabolism and exopolysaccharide production in genome analysis. Thus, cEPS of *B. bifidum* E3 were extracted and purified to explore its structural characteristics and antioxidant capacities. All EPSs were HePSs (primarily containing glucose, mannose, rhamnose, et al.). FTIR exhibited the existence of carboxyl and hydroxyl groups in all EPSs. At the same time, EPS-1, EPS-2, and EPS-3 have different free radical scavenging capacities, which are mainly related to their composition and structure. Therefore, EPS could be utilized as a promising natural antioxidant for application in functional foods. EPSs derived from *B. bifidum* E3 may provide a paradigm for future study of the structural and functional characteristics of EPS in antioxidants using genomics methods. 

## Figures and Tables

**Table 1:** Sugar transport system of *B. bifidum* E3.

Specificity	Locus_Tag	Product
Glucose	E3_00280	PTS system glucose-specific EIIA component
N-acetylglucosamine	E3_00281	PTS N-acetylglucosamine transporter subunit IIABC
Galactose	E3_01493	Galactose permease
Fructose	E3_01538	PTS system, fructose/glucose-specific IIABC
Fucose	E3_00598	Fucose permease
Lactose	E3_01046	Lactose permease Lactose transport system permease protein LacF
	E3_01493	
Sugar	E3_01045	multiple-sugar transport system permease protein
	E3_01046	ABC-type sugar transport system, permease component
	E3_01047, E3_01393	multiple-sugar transport system substrate-binding protein
	E3_01751	PTS sugar transporter subunit IIB

**Table 2:** Chemical compositions, monosaccharide compositions, and molecular weights of EPS-1, EPS-2, and EPS-3.

Sample	EPS-1	EPS-2	EPS-3
Yield (%)	29.33%	23.00%	10.67%
Neutral sugar (%)	90.53 ± 0.52 ^a^	89.16 ± 1.34 ^a^	72.47 ± 2.81 ^b^
Protein (%)	0.25 ± 0.09 ^b^	1.04 ± 0.10 ^b^	2.61 ± 0.63 ^a^
Uronic acid (%)	0.24 ± 0.08 ^c^	0.49 ± 0.06 ^b^	0.94 ± 0.07 ^a^
Sulphate (%)	1.65 ± 0.31 ^b^	2.26 ± 0.34 ^a^	2.33 ± 0.11 ^a^
Molecular weight (Da)	4.15 × 10^4^	3.67 × 10^4^	5.89 × 10^4^
Molar ratio	Man: Glu = 1:5.04	Rha: Man: Glu = 0.32: 1: 1.56	Rha: Man: GlcN: Gal: glu: GluA = 1.10: 1: 0.82: 1.05: 1.39: 0.68

^a–c^ Different letters in the same line indicated significant differences (*p* < 0.05).

## Data Availability

The data presented in this study are available on request from the corresponding author.
